# Supplementary material for: Precipitation extremes and depth-duration-frequency under internal climate variability
Source: Sci Rep. 2019 Jun 24;9:9112. doi: 10.1038/s41598-019-45673-3 (PMC6591321; doi:10.1038/s41598-019-45673-3)
Supplement: Supplementary file 1 — Supplementary Information Precipitation extremes and depth-duration-frequency under internal climate variability [file 41598_2019_45673_MOESM1_ESM.docx]

**Supplementary Information**

**Precipitation extremes and depth-duration-frequency under internal climate variability**

*Reducing the irreducible hydroclimate uncertainty*

Udit Bhatia, Auroop Ratan Ganguly

**SI: Precipitation extremes and depth-duration-frequency under internal climate variability**


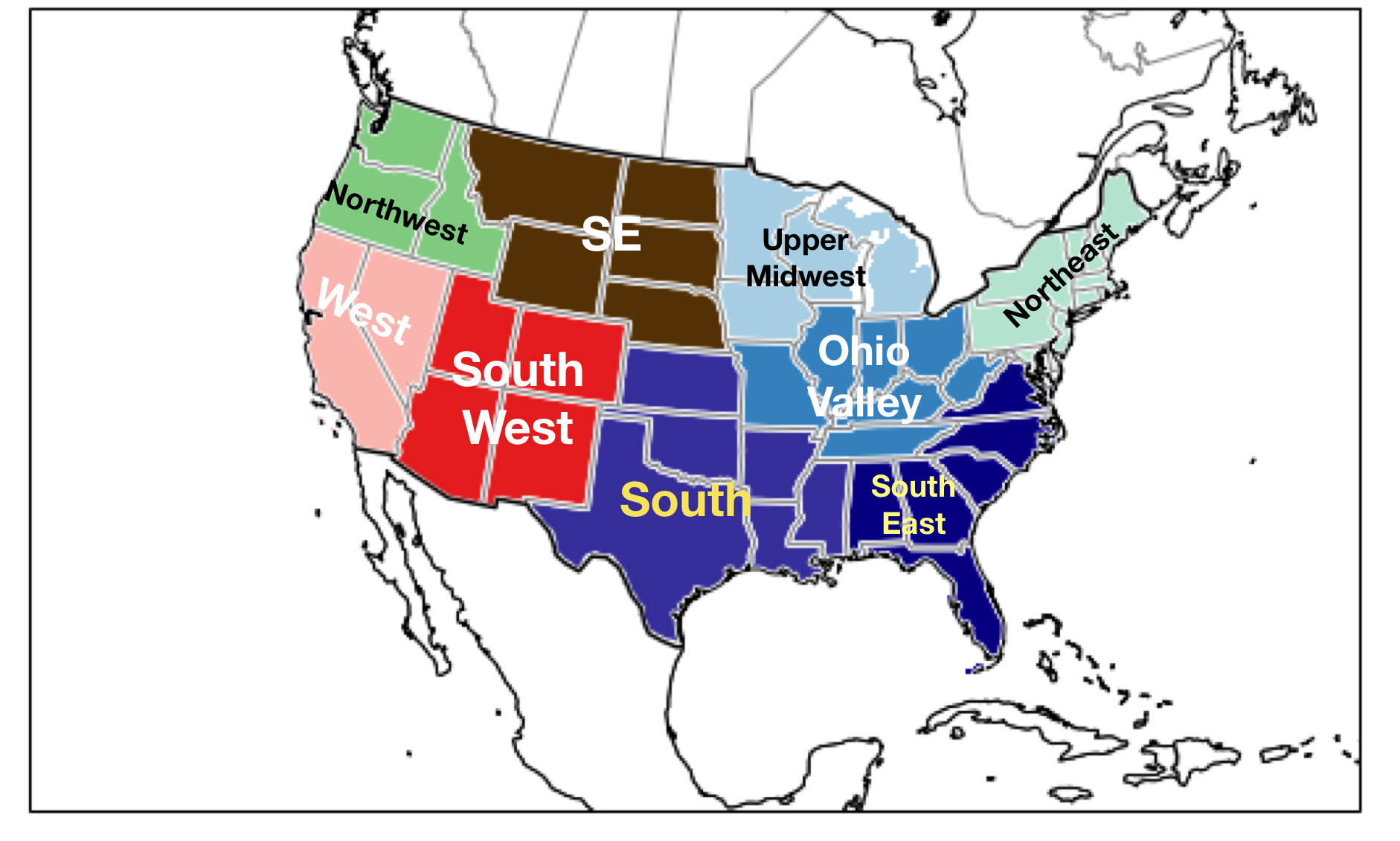
 **Fig. S1:** Nine climatically consistent regions within the contiguous United States which are useful for putting current climate anomalies into a historical perspective have been identified by National Centers for Environmental Information scientists. Figure above shows these 9 regions. Figure generated using Python 3.6 and shapely^1^


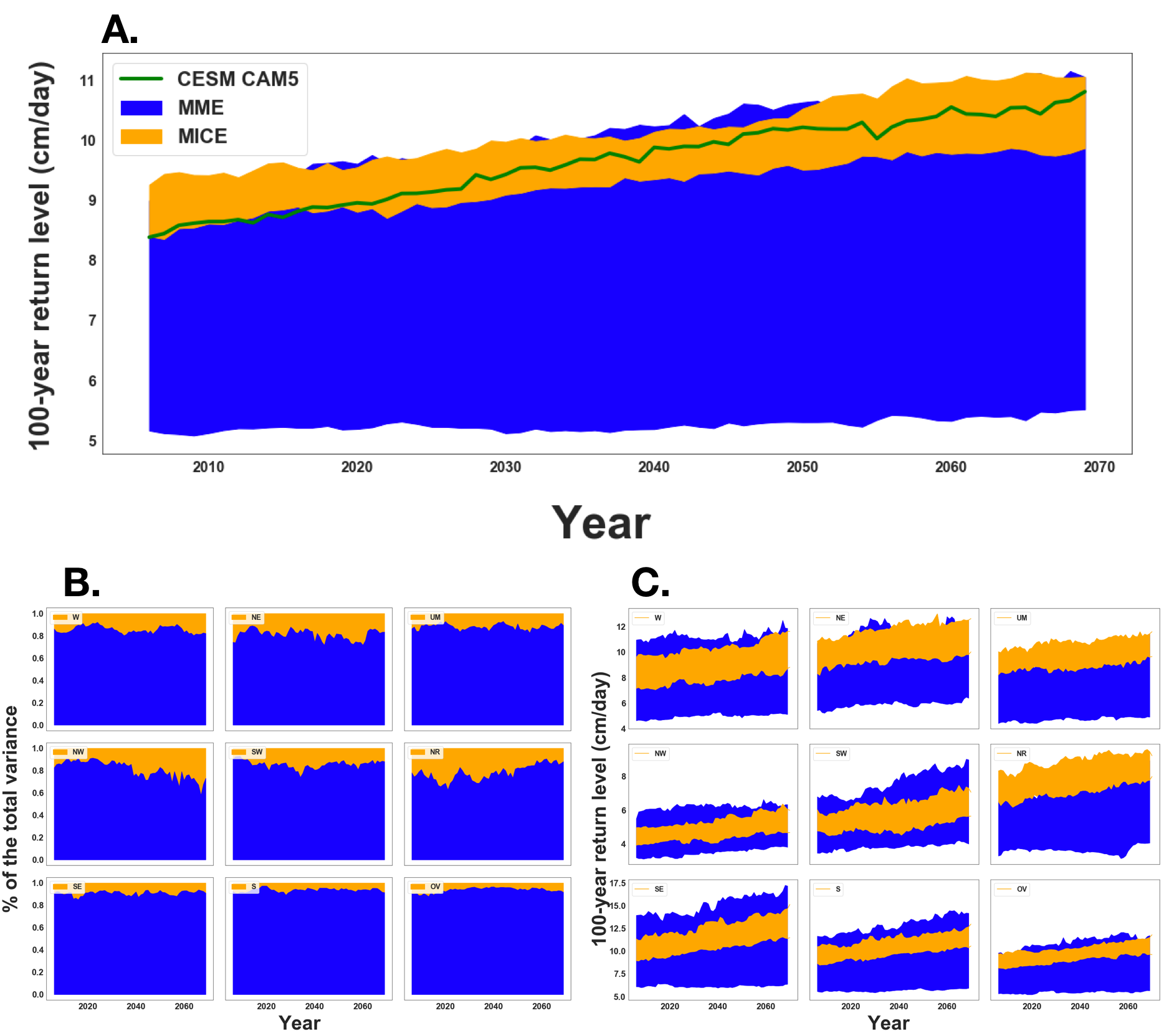


**Fig. S2: A.** Average estimates of 100-year return level (RLs) for entire CONUS using 30-year moving window beginning at 2006. Green line shows the RLs as obtained from CESM-CAM5, which is one of the ensembles in MME **B.** The ratio of MICE to MME variability is not significantly different across climate time horizons over this century for the 9 regions **C**. Same as figure A but for 9 climatological regions (See S1).

**Fig. S3**: Same as Figure 1B but for 9 regions shown in S1.
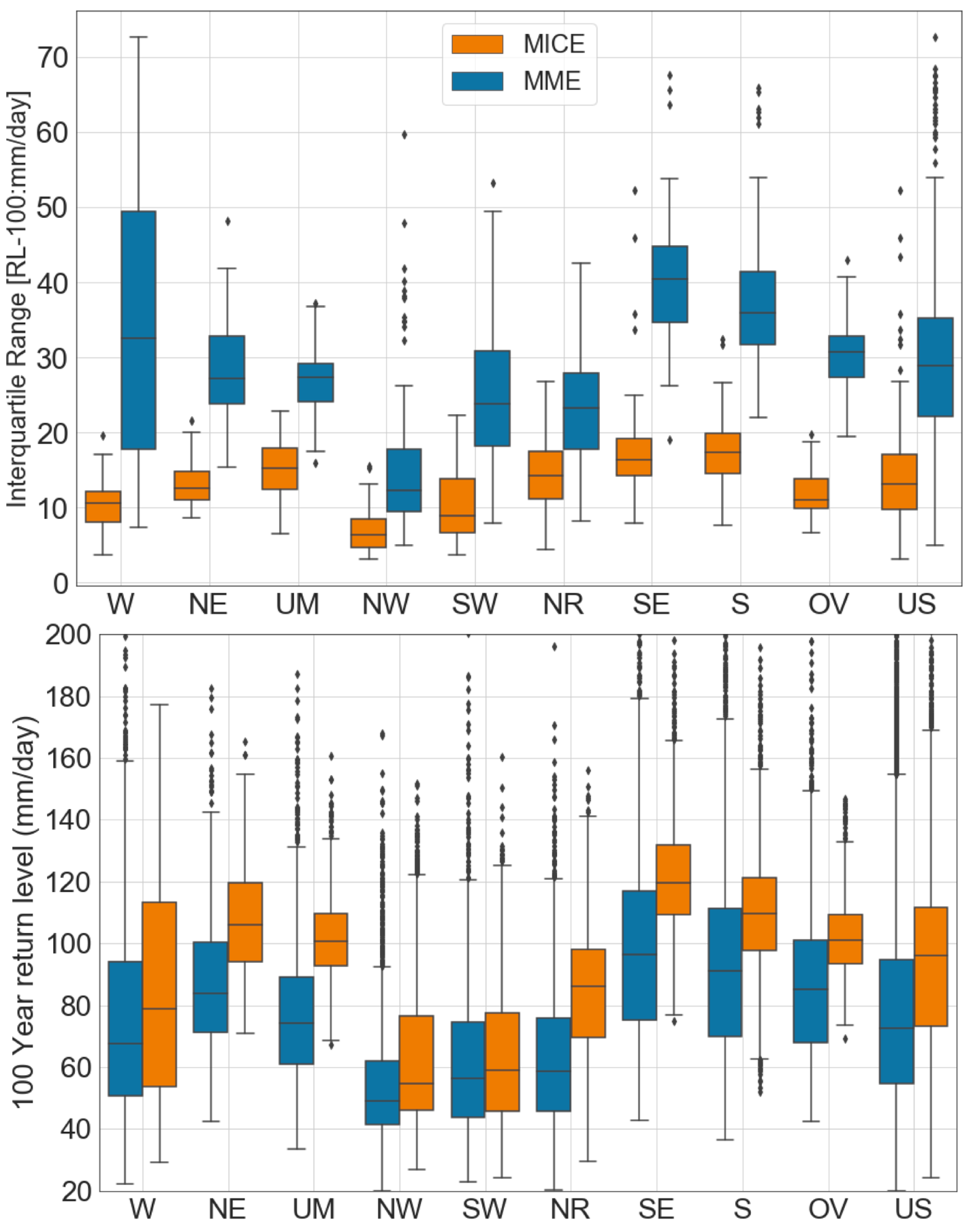


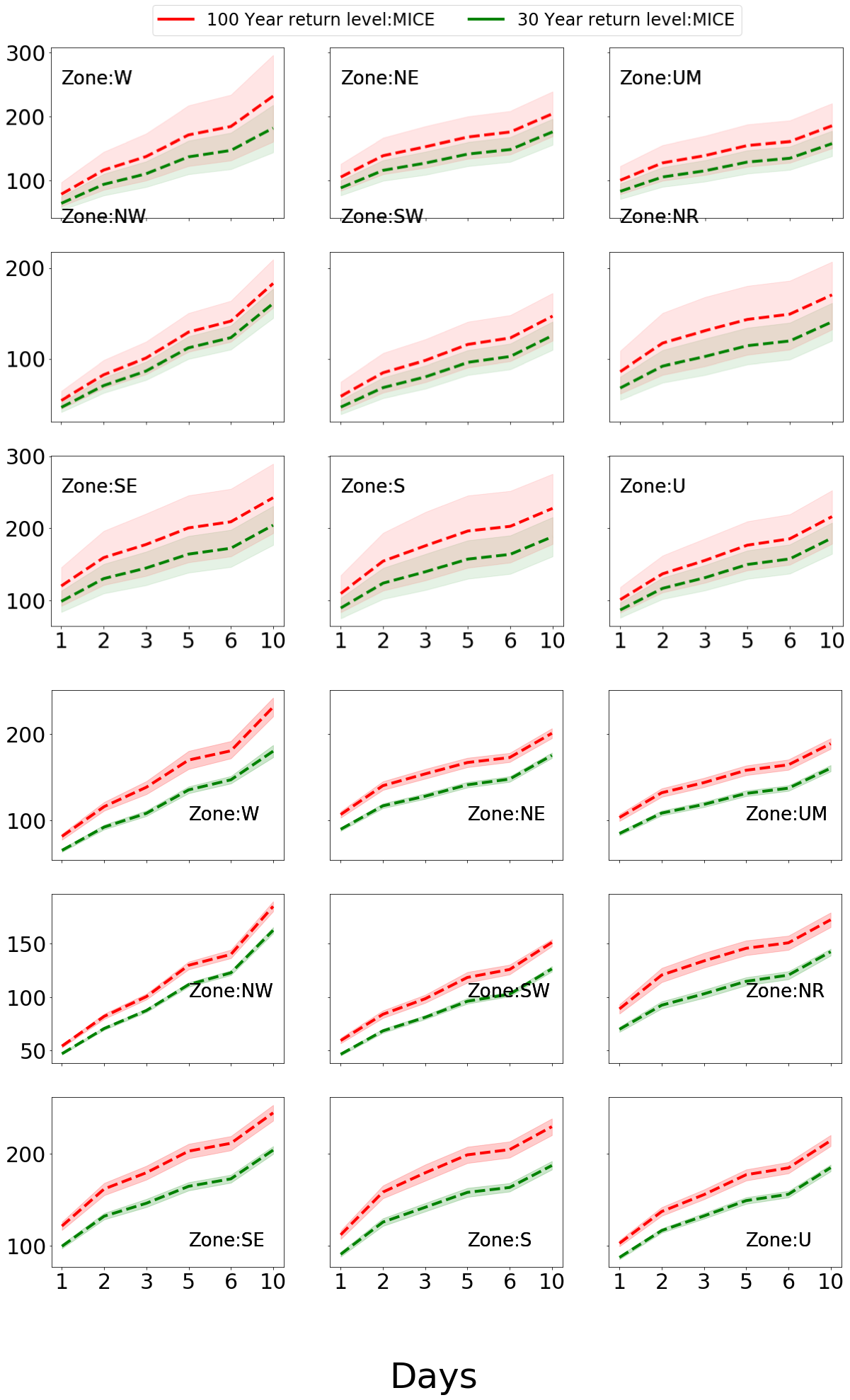


**Figure S3:** Same as Figure 3 but for all 9 regions shown in S1

**Table S1: List of CMIP5 models used in this study.**

| **Modeling Group** | **Model Name** |
| --- | --- |
| Commonwealth Scientific and Industrial Research Organization (CSIRO) and Bureau of Meteorology (BOM), Australia | ACCESS1-0 |
|  | ACCESS1-3 |
| Beijing Climate Center, China Meteorological Administration | bcc-csm1-1 |
|  | bcc-csm1-1-m |
| Beijing Normal University | BNU-ESM |
| Canadian Centre for Climate Modelling and Analysis | CanESM2 |
| National Center for Atmospheric Research | CCSM4 |
|  | CESM1-BGC |
|  | CESM1-CAM5 |
| Centro Euro-Mediterraneo per I Cambiamenti Climatici | CMCC-CESM |
|  | CMCC-CM |
|  | CMCC-CMS |
| Commonwealth Scientific and Industrial Research Organization in collaboration with Queensland Climate Change Centre of Excellence | CSIRO-Mk3-6-0 |
| EC-EARTH consortium | EC-EARTH |
| State Key Laboratory Numerical Modeling for Atmospheric Sciences and Geophysical Fluid Dynamics | FGOALS-g2 |
| NOAA Geophysical Fluid Dynamics Laboratory | GFDL-CM3 |
|  | GFDL-ESM2G |
|  | GFDL-ESM2M |
| Institute for Numerical Mathematics | inmcm4 |
| Institut Pierre-Simon Laplace | IPSL-CM5A-LR |
|  | IPSL-CM5A-MR |
|  | IPSL-CM5B-LR |
| Atmosphere and Ocean Research Institute (The University of Tokyo), National Institute for Environmental Studies, and Japan Agency for Marine-Earth Science and Technology | MIROC-ESM |
|  | MIROC-ESM-CHEM |
|  | MIROC-5 |
| Max Planck Institute for Meteorology | MPI-ESM-LR |
|  | MPI-ESM-MR |
| Meteorological Research Institute | MRI-CGCM3 |
| Norwegian Climate Centre | NorESM1-M |

**References:**

1. Gillies S. and others. Shapely: manipulation and analysis of geometric objects, 2007--, URL: https://github.com/Toblerity/Shapely
